# Association of *qacA/B* and *smr* Carriage with Staphylococcus aureus Survival following Exposure to Antiseptics in an *Ex Vivo* Venous Catheter Disinfection Model

**DOI:** 10.1128/spectrum.03333-22

**Published:** 2023-03-02

**Authors:** J. Chase McNeil, Lauren M. Sommer, Jesus G. Vallejo, Kristina G. Hulten, Sheldon L. Kaplan

**Affiliations:** a Department of Pediatrics, Division of Infectious Diseases, Baylor College of Medicine, Houston, Texas, USA; b Texas Children’s Hospital, Houston, Texas, USA; University of Calgary

**Keywords:** *smr*, *qacA/B*, disinfection, chlorhexidine gluconate, *Staphylococcus aureus*, pediatric

## Abstract

Many health care centers have reported an association between Staphylococcus aureus isolates bearing efflux pump genes and an elevated MIC/minimal bactericidal concentration (MBC) to chlorhexidine gluconate (CHG) and other antiseptics. The significance of these organisms is uncertain, given that their MIC/MBC is typically far lower than the CHG concentration in most commercial preparations. We sought to evaluate the relationship between carriage of the efflux pump genes *qacA/B* and *smr* in S. aureus and the efficacy of CHG-based antisepsis in a venous catheter disinfection model. S. aureus isolates with and without *smr* and/or *qacA/B* were utilized. The CHG MICs were determined. Venous catheter hubs were inoculated and exposed to CHG, isopropanol, and CHG-isopropanol combinations. The microbiocidal effect was calculated as the percent reduction in CFU following exposure to the antiseptic relative to the control. The *qacA/B-* and *smr*-positive isolates had modest elevations in the CHG MIC_90_ compared to the *qacA/B-* and *smr*-negative isolates (0.125 mcg/ml vs. 0.06 mcg/ml, respectively). However, the CHG microbiocidal effect was significantly lower for *qacA/B*- and/or *smr*-positive strains than for susceptible isolates, even when the isolates were exposed to CHG concentrations up to 400 μg/mL (0.04%); this finding was most notable for isolates bearing both *qacA/B* and *smr* (89.3% versus 99.9% for the *qacA/B-* and *smr*-negative isolates; *P* = 0.04). Reductions in the median microbiocidal effect were also observed when these *qacA/B-* and *smr*-positive isolates were exposed to a solution of 400 μg/mL (0.04%) CHG and 70% isopropanol (89.5% versus 100% for the *qacA/B-* and *smr*-negative isolates; *P* = 0.002). *qacA/B*- and *smr-*positive S. aureus isolates have a survival advantage in the presence of CHG concentrations exceeding the MIC. These data suggest that traditional MIC/MBC testing may underestimate the ability of these organisms to resist the effects of CHG.

**IMPORTANCE** Antiseptic agents, including chlorhexidine gluconate (CHG), are commonly utilized in the health care environment to reduce rates of health care-associated infections. A number of efflux pump genes, including *smr* and *qacA/B*, have been reported in Staphylococcus aureus isolates that are associated with higher MICs and minimum bactericidal concentrations (MBCs) to CHG. Several health care centers have reported an increase in the prevalence of these S. aureus strains following an escalation of CHG use in the hospital environment. The clinical significance of these organisms, however, is uncertain, given that the CHG MIC/MBC is far below the concentration in commercial preparations. We present the results of a novel surface disinfection assay utilizing venous catheter hubs. We found that *qacA/B*-positive and *smr*-positive S. aureus isolates resist killing by CHG at concentrations far exceeding the MIC/MBC in our model. These findings highlight that traditional MIC/MBC testing is insufficient to evaluate susceptibility to antimicrobials acting on medical devices.

## INTRODUCTION

Gram-positive bacteria are the principal organisms causing health care-associated infections (HAIs) in both adults and children (1–3). While substantial progress has been made in reducing the rates of these infections (4), HAIs continue to place significant burdens on patients. Staphylococcus aureus ranks among the most common and clinically important contributors to HAIs (5, 6).

One potential strategy to decrease the frequency of HAIs utilizes topical antiseptic agents; among the most commonly used is chlorhexidine gluconate (CHG). CHG is a biguanide antiseptic that nonspecifically disrupts the bacterial cell wall and interferes with the cellular membrane potential of numerous microorganisms, including Gram-positive and Gram-negative bacteria as well as yeasts (7). CHG-containing products in a variety of formulations have been shown to decrease the rates of HAIs (8–11). Such strategies have been endorsed by the Centers for Disease Control and Prevention (CDC) and the Infectious Diseases Society of America (IDSA) for the prevention of HAIs (12–14).

While there is a clear benefit from these agents, any attempt to pharmacologically modify the natural flora has the potential for the evolution of reduced susceptibility over time. A number of efflux pump genes have been reported in Gram-positive pathogens that are associated with higher MICs and minimum bactericidal concentrations (MBCs) to CHG and other antiseptics (such as benzalkonium chloride and cetrimide) (15–18). In S. aureus, the plasmid-borne *smr* and the *qacA/B* gene complexes have been most commonly implicated, with such organisms often being termed antiseptic tolerant (19). QacA and QacB belong to the major facilitator superfamily of proton-dependent efflux pumps and confer reduced susceptibility to quaternary ammonium compounds, ethidium bromide, and biguanides (e.g., CHG), as well as fluoroquinolones (20, 21). QacA and QacB differ by only six amino acids; they have a similar substrate profile and are often referred to collectively in the literature as *qacA/B*. The *smr* (staphylococcal multidrug resistance) gene encodes a dimeric efflux pump with similar activity, albeit the reduction in susceptibility to CHG is less pronounced than in *qacA/B*-positive strains (21–23). A number of investigators have reported an increase in the incidence of such S. aureus strains following the widespread use of CHG in hospital units (24–26) and, more rarely, following exposure to CHG outside the hospital setting (27). A British study reported the implementation of CHG bathing in an intensive care unit with both endemic methicillin-resistant S. aureus (MRSA) and a *qacA/B*-positive MRSA epidemic strain. These investigators noted a dramatic decline in patient colonization with endemic MRSA strains but an increase in colonization with the *qacA/B*-positive epidemic strain following CHG use (28). Currently, in some centers in the United States, >50% of hospital-acquired S. aureus bloodstream isolates carry either *qacA/B* or *smr* (29).

While *qacA/B*- and *smr*-positive S. aureus strains have been associated with elevated MIC/MBC to antiseptics, controversy exists regarding the clinical implications of these organisms, given that the MIC/MBC still remain far lower than the concentrations of agents in many commercially available preparations (in the case of CHG, as high as 40,000 μg/mL [4%]). Even among *smr*- and/or *qacA/B*-positive clinical isolates, variability in the MIC/MBC has been reported, further clouding this issue. The ideal method to determine antiseptic susceptibility is debatable, with some authors suggesting that susceptibility testing should closely mimic clinical scenarios in which the antimicrobial agent of interest is used (30, 31). This is relevant as antiseptics are applied to the surfaces of medical equipment or topically to patients and achieve high concentrations locally, rather than being administered systemically and distributed throughout the host. Thus, traditional MIC/MBC testing may not accurately reflect the efficacy of antiseptic agents working on the surface of the body or of medical devices, such as central venous catheters. Central line-associated bloodstream infections are among the most prominent health care-associated S. aureus infections (32), and one of the primary reasons that CHG is used in clinical practice is for the prevention of these infections. Such infections most commonly occur through either extraluminal contamination of the catheter hub or the spread of colonizing organisms from adjacent skin. In hospitalized patients with central venous catheters, bathing with CHG has been associated with reduced rates of bloodstream infections (8). Furthermore, CHG and CHG-alcohol solutions are commonly used in central venous catheter insertion and maintenance procedures, as well as for cleansing of the catheter hub prior to the administration of medications/fluids (11, 13). Therefore, we sought to evaluate the ability of *smr*- and *qacA/B*-positive S. aureus clinical strains to resist disinfectant killing in an *ex vivo* venous catheter disinfection model. In these assays, we assessed both logarithmic and linear measures of antiseptic efficacy and report the results in terms of log reduction (33) and microbiocidal effect (ME) (34). While not a true mechanistic investigation, the purpose of this study is 2-fold: (i) to assess the relationship between the CHG MIC/MBC, carriage of *qacA/B* and/or *smr* in S. aureus isolates, and the ability to resist surface disinfection and (ii) to examine the heterogeneity in disinfectant susceptibility among *smr*- and *qacA/B*-positive S. aureus isolates.

## RESULTS

The characteristics of the tested S. aureus clinical isolates are summarized in Table S1 in the supplemental material. Four random clinical strains were used to examine the ability to recover staphylococci from the catheters in the absence of antiseptic; the median concentration of organisms recovered was 522,500 CFU/mL (interquartile range [IQR], 402,500 to 682,500).

**FIG 1:**
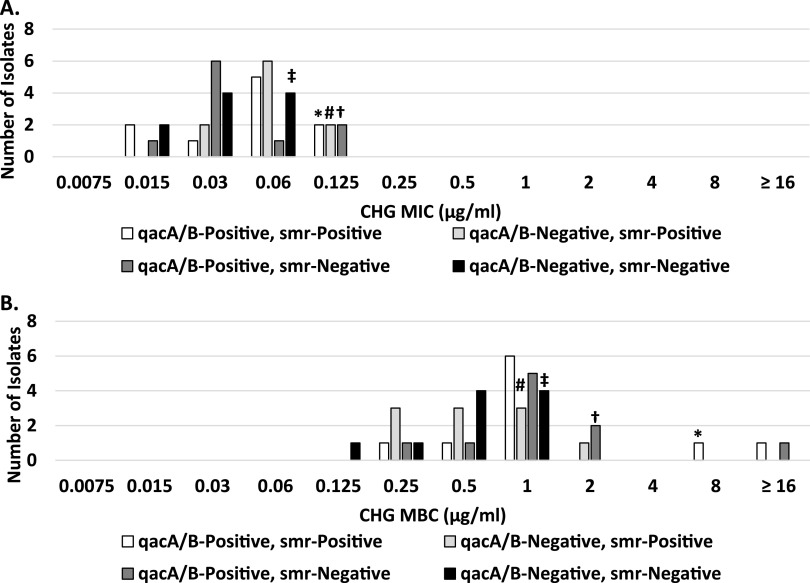
Chlorhexidine broth dilution MICs (A) and minimum bactericidal concentrations (MBCs) (B). The MIC_90_ and MBC_90_ values are indicated for each genotype: *, *qacA/B* positive, *smr* positive; #, *qacA/B* negative, *smr* positive; †, *qacA/B* positive, *smr* negative; ‡, *qacA/B* negative, *smr* negative.

Isolates bearing *qacA/B* and/or *smr* genes had a slightly higher CHG MIC compared to those isolates lacking these genes (MIC_90_, 0.125 versus 0.06 μg/mL, respectively; *P* = 0.12), as well as slight elevations in MBCs (MBC_90_, 2 versus 1 μg/mL, respectively; *P* = 0.1) (Fig. 1A and B).

### Microbiocidal effect and log reduction of CHG.

**FIG 2:**
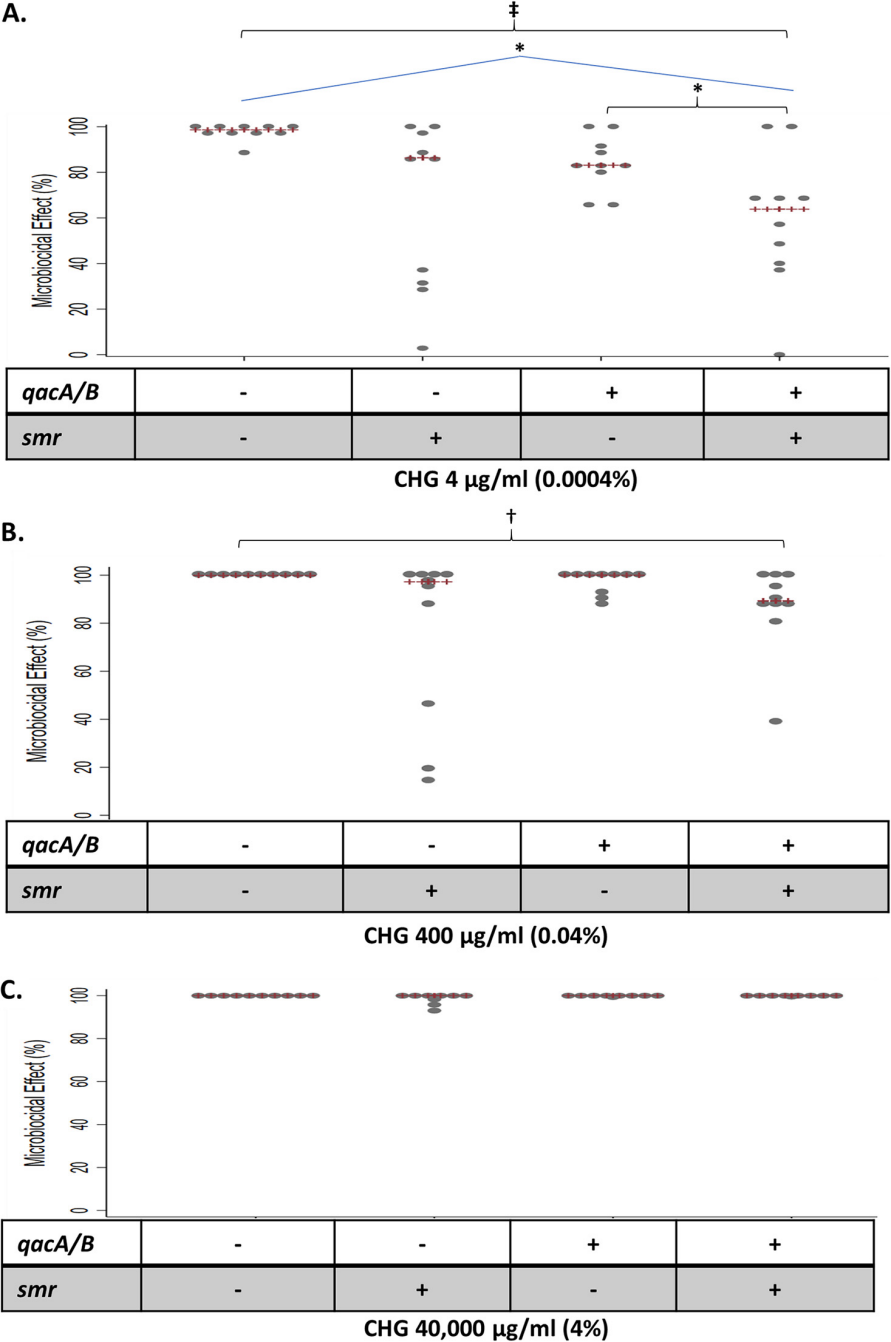
Dot plots displaying the results of catheter disinfection assays depicting the microbiocidal effect (ME) of 4 μg/mL CHG (0.0004%) (A), 400 μg/mL CHG (0.04%) (B), and 40,000 μg/mL (4%) (C). The red lines (+++) correspond to the median ME. Two-way comparisons were performed for all combinations of genotypes (Wilcoxon rank sum test), and four-way comparisons were performed across all genotypes (Kruskal-Wallis test). Statistically significant findings are indicated as follows: *, *P* = 0.01 to 0.05 (rank sum—2 category comparison); **, *P* < 0.01 (rank sum—2 category comparison); †, *P* = 0.01 to 0.05 (Kruskal-Wallis—4 category comparison); ‡, *P* < 0.01 (Kruskal-Wallis—4 category comparison).

The ability of CHG alone to eradicate bacteria adhered to the catheters was examined (Fig. 2A to C). At a CHG concentration of 4 μg/mL (0.0004%), significant differences in the logarithmic reduction in CFU across genotypes was observed (ranging from a median log reduction of 1.1 [IQR, 0.5 to 1.8] among *qacA/B*-positive, *smr*-positive isolates to 2.2 [IQR, 1.7 to 2.9] among *qacA/B*-negative, *smr*-negative-isolates; *P* = 0.01). The ME was calculated as the percent reduction in CFU following exposure to the antiseptic relative to the control. The median percent ME ranged from 55.95% (IQR, 39.3% to 69.4%) for *qacA/B*-positive, *smr*-positive isolates to 98.7% (IQR, 98.1% to 99.4%) for *qacA/B*-negative, *smr*-negative-isolates (*P* = 0.004) (Fig. 2A). Using a CHG concentration of 400 μg/mL (0.04%), the ME was reduced in *qacA/B*-positive, *smr*-positive isolates (89.3%; IQR, 84% to 99.8%) compared to *qacA/B*-negative, *smr*-negative isolates (99.9%; IQR, 99.8% to 99.96%; *P* = 0.04) (Fig. 2B); there was no significant difference in log reduction at this concentration. At the highest CHG concentration assessed (40,000 μg/mL [4%]), no significant differences were observed in the ME (Fig. 2C).

There was no statistically significant correlation between the CHG MBC and ME (correlation between MBC and ME at 400 μg/mL CHG; Spearman’s *r* = −0.14; *P* = 0.1). The three isolates with a CHG MBC of ≥8 μg/mL (two isolates were *qacA/B* positive, *smr* positive and one was *qacA/B* positive, *smr* negative), however, we associated with low ME values when tested with 400 μg/mL CHG (ranging from 14.4% to 79.9%).

### Microbiocidal effect of isopropanol.

The ability of isopropanol alone to eradicate bacteria in this catheter model was assessed as a control. The *qacA/B* and *smr* genes are not believed to confer reduced susceptibility to alcohols, and thus we expected similar median MEs across the strain types studied. While variability in the ME existed, no significant difference in the median MEs was observed between isolates with and without *qacA/B* and/or *smr* in the presence of 0.7% to 70% (vol/vol) isopropanol.

### Microbiocidal effect of combination antiseptic solutions.

**FIG 3:**
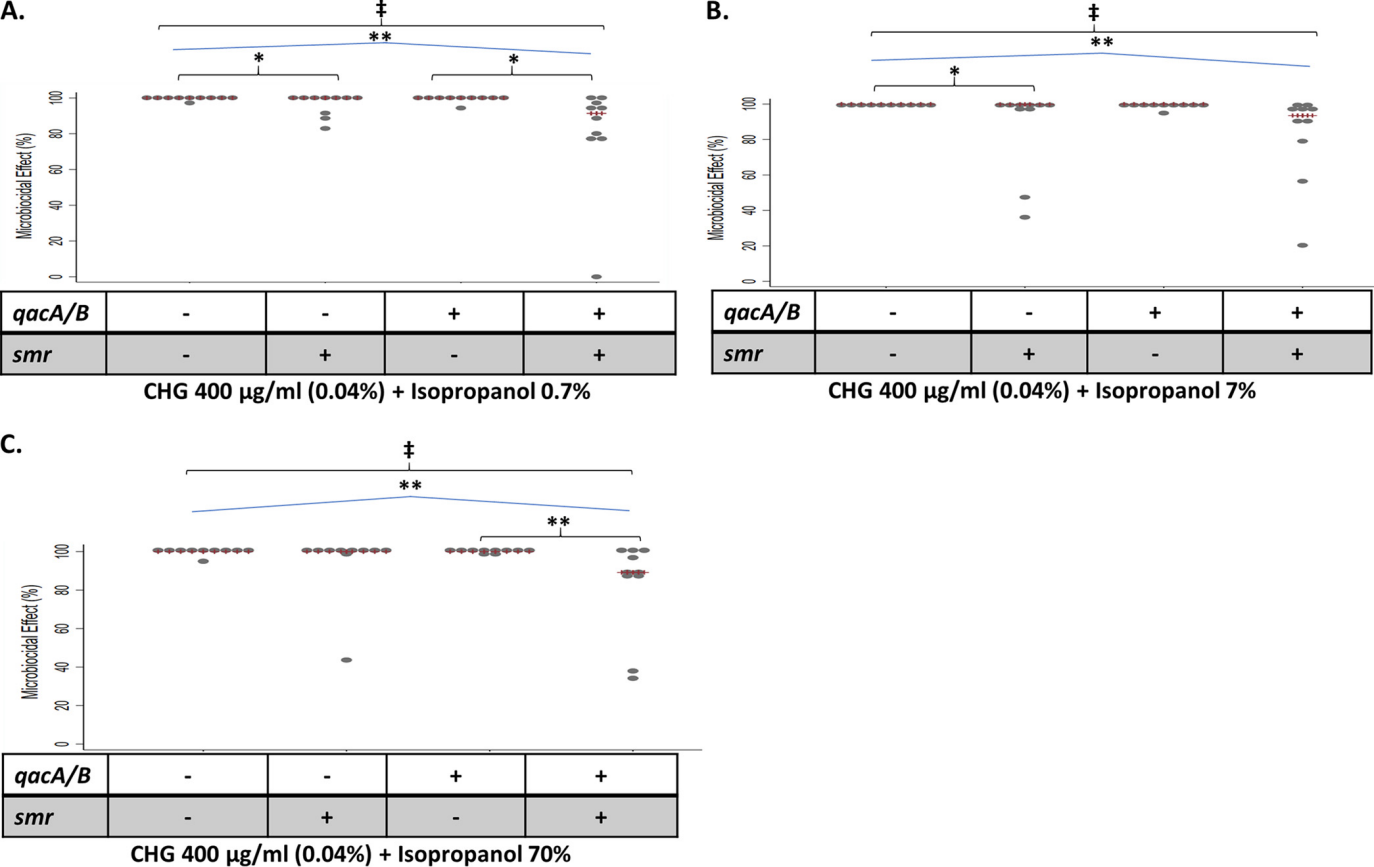
Results of catheter disinfection assays depicting the microbiocidal effect (ME) of 400 μg/mL CHG (0.04%) plus 0.7% isopropanol (A), 400 μg/mL CHG plus 7% isopropanol (B), and 400 μg/mL CHG plus 70% isopropanol (C).

Similar experiments were conducted with combinations of CHG at 400 μg/mL (0.04%) and isopropanol. Statistically significant reductions in the ME were observed for *qacA/B*-positive, *smr*-positive isolates compared to *qacA/B*-negative, *smr*-negative isolates or isolates bearing only one of these genes at isopropanol concentrations of up to 70% (vol/vol) (Fig. 3A to C). When the CHG concentration was increased to 40,000 μg/mL (4%) and combined with isopropanol, no organisms were recovered at any of the isopropanol concentrations tested.

The efficacy of 5,000 μg/mL CHG (equivalent of 0.5% CHG) plus isopropanol for catheter disinfection was examined for *qacA/B*-positive, *smr*-positive isolates and *qacA/B*-negative, *smr*-negative isolates. A small but statistically significant reduction in the ME was noted among *qacA/B*-positive, *smr*-positive isolates exposed to 5,000 μg/mL CHG combined with 0.7% isopropanol (Fig. S1).

### Relationship of Serial CHG Exposure and Microbiocidal Effect.

The ME of repeated exposure to 400 μg/mL CHG was assessed in one *qacA/B*-positive, *smr*-positive strain and one strain negative for both these genes. After three passages, the ME was >99% for both isolates. There was no significant change in MIC or MBC observed.

## DISCUSSION

Previous investigators have examined the capability of antiseptics to decontaminate flat surfaces to which microorganisms have been adhered (31, 34, 35). Notably, such studies may not recapitulate clinical scenarios well; both human skin and medical devices may have irregular surfaces, leading to nonuniform distribution of an antiseptic. Such hypotheses are indirectly supported by clinical series demonstrating decolonization failure and breakthrough infections in the presence of antiseptic-tolerant organisms, despite high CHG concentrations being used (28, 36). The growth of microorganisms on solid-phase media or surfaces may allow for the detection of antiseptic-resistant or -tolerant subpopulations of bacteria (37), which may manifest in the clinical setting, as is the case with some systemic antimicrobials (e.g., vancomycin-heteroresistant S. aureus [hVISA]) (38). To address such concerns, we developed an *ex vivo* venous catheter disinfection model, using modifications of existing techniques.

Among strains bearing both *qacA/B*-and *smr*, we observed a statistically significant difference in survival following exposure to CHG concentrations of up to 400 μg/mL (0.04%), >3,000-fold in excess of the MIC_90_. Such findings underscore the limitations of traditional MIC/MBC in determining susceptibility to antiseptics. Our findings are in agreement with a recent study in which *qacA/B*-positive S. aureus strains had a survival advantage compared to *qacA/B*-negative strains in time-kill assays with 0.5% (5,000 μg/mL) CHG (39). As would be expected, no survival benefit was observed in antiseptic-tolerant staphylococci when exposed to isopropanol alone in our study. However, a small but statistically significant survival benefit was observed in *qacA/B*-positive, *smr*-positive strains when exposed to CHG-isopropanol combinations, albeit the MEs were much higher than those associated with CHG exposure alone and there was a similar log reduction. These data suggest that the survival advantage in these strains may be partially, but not completely, obviated by the addition of alcohols to CHG-containing preparations. Moreover, given the survival advantage of *qacA/B*-positive S. aureus strains in the face of CHG concentrations in the low range of those clinically used (5,000 μg/mL [0.5%]), our findings highlight the potential clinical significance of these organisms. Furthermore, it is conceivable that in clinical practice there may be differential eradication of S. aureus on the external surfaces of venous catheters after CHG application, resulting in the survival of *qacA/B*-/*smr*-positive subpopulations. Such a mechanism may potentially explain the relative increase in *qacA/B*-positive S. aureus infections following CHG use in some hospital units (24, 25, 28). Additionally, the antiseptic concentration seen at the patient level may be far less than that contained in commercial preparations. In a study of patients receiving bathing with 2% (20,000 μg/mL) CHG wipes, the concentration of CHG recovered from the skin immediately after bathing was <300 μg/mL in 80% of samples (40). In total, these findings emphasize the potential limitations of current prevention strategies.

It is worth acknowledging the observed variability in ME within antiseptic-tolerant isolates. Antiseptic-tolerant S. aureus have distinct genetic backgrounds compared to antiseptic-susceptible strains; *qacA/B*-positive, *smr*-positive S. aureus strains more often belong to *agr* IV and appear to be unrelated to the common community-acquired S. aureus lineage in the United States (sequence type 8 [ST8], strain USA300) (41). It is conceivable that at least a component of the reduced antiseptic susceptibility may be related to intrinsic properties of the individual strain rather than the action of the efflux pump genes themselves. Furthermore, previous studies have reported differential levels of expression in S. aureus of multidrug resistance efflux pumps, including *qacA/B* (42). Future work may need to focus on measuring expression of the *qacA/B*/*smr* gene products and their relationships to the ME and/or MIC. Alternatively, the range of MEs found may represent errors introduced by the irregular surface of venous catheters (with ridges and grooves), as it was difficult to guarantee uniform antiseptic exposure across the catheter, albeit similar situations would conceivably arise with the application of antiseptic to venous catheters in clinical practice.

There are additional limitations to this work which should be acknowledged. Foremost, these results are from an internally developed assay which has not been externally validated. While we frequently observed linear reductions in CFU in *qacA/B*- and/or *smr*-positive strains (as manifested by a reduced ME), logarithmic decreases in organisms were not consistently observed. The tendency of S. aureus cells to grow in clusters may have impacted the ability to consistently define organism numbers, potentially contributing to the variability in ME (43). In addition, the use of clinical S. aureus isolates, rather than well-characterized laboratory strains, introduces the potential for bias secondary to unknown factors in these strains. However, the use of clinical strains, many of which were obtained from HAIs, provides an element of “real world” utility to the model. One of the primary goals in developing this model was to illustrate the limitations of traditional MIC testing in evaluating antiseptic susceptibility, rather than performing a true mechanistic investigation. Furthermore, the utilization of a commonly used medical device (venous catheter), clinically meaningful antiseptic concentrations, combinations of antiseptic solutions, and the attempt to replicate bedside infection control practices (direct application of the antiseptic, allowing the antiseptic to dry, etc.) provide additional elements of clinical relevance to this laboratory assay. Notably, the inoculum of bacteria used and applied directly to the catheter hub likely far exceeds that which contributes to typical catheter colonization/infection. While the organism burden on the external surfaces of venous catheters present in patients is not well described, previous IDSA practice guidelines have defined catheter colonization as the growth of >10^2^ CFU from sonicated catheter tips (44). It is conceivable that with a lower bacterial inoculum, small differences in the ME would have been less apparent. The inoculum used in our experiments was, however, consistent with that used in previous surface disinfection research (34). The period of incubation with antiseptic and mechanical agitation (5 min) far exceeded the recommended minimum 5 to 30 s period of “hub scrub” used in clinical practice (45, 46). If anything, however, these interventions may have falsely elevated the ME and thus the degree of antiseptic tolerance may not have been fully appreciated. Additionally, the brief incubation of the catheters in broth following disinfection may have allowed for surviving organisms to proliferate and perhaps impacted the ME determinations; however, this would be expected to impact all genotypes in a similar fashion.

In conclusion, we present a clinically relevant *ex vivo* venous catheter disinfection model. Using this model, *qacA/B*-positive, *smr*-positive S. aureus strains have a survival advantage in the presence of CHG concentrations up to 5,000 μg/mL (0.5%), far in excess of the MIC_90_. Thus, traditional MIC/MBC testing likely underestimates the ability of these strains to survive antiseptic exposure. These findings suggest that some clinically used CHG solutions may fail to disinfect *qacA/B*-/*smr*-positive S. aureus colonized/contaminated catheters, medical devices, or conceivably, patient skin.

## MATERIALS AND METHODS

### Isolates.

S. aureus isolates were identified from a previously published study conducted at Texas Children’s Hospital (TCH) (47). Briefly, these isolates were taken from a study for which all S. aureus isolates identified by the clinical microbiology laboratory at TCH in 2014 were eligible and screened for *smr*- and *qacA/B* using PCR and previously published primers (41). All isolates were stored at −80°C in horse blood at the Edward O. Mason Infectious Diseases Research Laboratory at TCH immediately after isolation; the isolates were not serially passaged. Ten clinical isolates each of the following genotypes were selected at random from the previously characterized subset: (i) *qacA/B*-positive, *smr*-positive; (ii) *qacA/B*-positive, *smr*-negative; (iii) *qacA/B*-negative, *smr*-positive; and (iv) *qacA/B*-negative, *smr*-negative. Thus, 40 isolates total (10 of each genotype) were examined in this study.

### Antiseptic solutions.

Chlorhexidine gluconate (40,000 μg/mL; Sigma-Aldrich) and isopropanol (70%; Sigma-Aldrich) solutions were used in the disinfection experiments and diluted in sterile water to achieve the desired concentrations. The chlorhexidine concentrations ranged from 4 to 40,000 μg/mL (0.0004% to 4%) in 100-fold dilutions; the isopropanol concentrations ranged from 0.7% to 70% (vol/vol) in 10-fold dilutions. The high concentration of CHG utilized (40,000 μg/mL) was chosen since this is the maximum concentration contained in commercially available CHG wipes (4%). While the lowest concentrations of antiseptic are not typically utilized clinically, the various dilutions of antiseptic were intended to investigate a dose-response relationship between organism survival and the antiseptic concentration. In addition, combination solutions containing both CHG and isopropanol at various concentrations were examined.

### *Ex vivo* venous catheter hub disinfection model.

The ability of antiseptic to eradicate bacteria from venous catheters was examined in a modification of previously described surface disinfection assays (31, 34, 35). Following overnight growth on blood agar plates, a bacterial suspension was prepared in sterile saline to a concentration of 1.5 × 10^8^ CFU/mL. Ten microliters of this suspension (approximately 1.5 × 10^6^ CFU) (34) was pipetted directly onto the external surface of venous catheter hubs (Ultrasite needleless injection system; Braun Healthcare; product code US1320), similar to those commonly used for the intravenous administration of medications. The bacterial suspension was allowed to dry on the catheters, which were incubated in a sterile laminar flow hood at room temperature for 2 h. Four catheters were used per isolate with each antiseptic agent or combination tested; the catheters were not reused in these studies. Following inoculation, three catheters were exposed to progressively higher concentrations of antiseptic solution, while the fourth catheter was used as a control and not exposed to antiseptic. One hundred microliters of antiseptic preparation was applied externally to the venous catheter hubs using a sterile technique, followed by mechanical agitation at 150 rpm in a shaking incubator at room temperature for 5 min. The catheters were then allowed to dry in a sterile hood for 5 min. Following this, a previously described neutralizer solution (0.75% phosphatidyl choline and 5% Tween 80) (34) was applied to the catheter hubs; 900 μL was applied to the catheters that received antiseptic and 1,000 μL to the control catheters, which did not receive antiseptic (35). Following drying, the catheters were then placed in 10 mL of tryptic soy broth, mechanically agitated in a shaking incubator for 5 min, and then incubated for 1 h at 37°C. The purpose of this step was to disperse surviving bacteria adhered to the catheters. Aliquots (10 μL) of each broth culture were then serially diluted and inoculated onto blood agar plates in duplicate; colony counts were performed after 24 h of incubation. An initial set of experiments was performed using four random strains to assay the recovery of organisms from the catheters without the application of antiseptics.

In the above-described model, both logarithmic and linear measures of antiseptic efficacy were assessed. Historically, log_10_ reductions in organisms were used to assess the efficacy of antiseptics (48); however, such an approach may not highlight subtle differences in relative susceptibility (34). The log_10_ reduction in CFU was reported as the log_10_ CFU of the control minus the log_10_ CFU of the experimental condition (33). Additionally, the microbiocidal effect (ME) was measured, which was calculated as the percent reduction in CFU following exposure to an antiseptic relative to that of the control (34) and can be expressed as follows: ([CFU control – CFU experimental]/CFU control) × 100%. An ME of 100% represents the complete eradication of all bacteria. The ME was used to adjust for potential variability in colony recovery across control experiments.

Antiseptic solutions were also used in traditional broth macrodilution assays (49) in Mueller-Hinton broth to determine the CHG MICs for all isolates. The MICs were read by visual inspection following overnight incubation at 37°C; at this time, aliquots of each dilution were streaked onto blood agar plates and allowed to incubate overnight to determine the MBC in accordance with Clinical and Laboratory Standards Institute guidance (49). For a subset of isolates, the surviving colonies after CHG exposure in the catheter disinfection experiments were then subjected to additional catheter disinfection experiments to assess the impact of repeated CHG exposure on the ME, as well as the MIC and MBC.

### Statistical analyses.

ME and log reduction data were recorded for all isolates; the laboratory technicians performing the disinfection assays were blinded to the bacterial genotype. Comparisons were made in ME/log reduction across the categories of bacterial genotypes using Kruskal-Wallis (comparing all four genotypes) and Wilcoxon rank sum (comparing two genotypes at a time) tests, as appropriate; data from all isolates were included in these analyses. The MIC values were compared using Fisher’s exact test. The relationship between the ME and MBC was examined using the Spearman correlation test. *P* values of <0.05 were considered statistically significant. STATA ver. 15 was used in the statistical analyses.
