# Supplementary material for: Association of qacA/B and smr Carriage with Staphylococcus aureus Survival following Exposure to Antiseptics in an Ex Vivo Venous Catheter Disinfection Model
Source: Microbiol Spectr. 2023 Mar 2;11(2):e03333-22. doi: 10.1128/spectrum.03333-22 (PMC10100659; doi:10.1128/spectrum.03333-22)
Supplement: Supplemental file 1 — Supplemental material. Download spectrum.03333-22-s0001.pdf, PDF file, 0.3 MB [file spectrum.03333-22-s0001.pdf]

Supplemental Table 1. General Characteristics of Isolates

Genotype	*qacA/B*-Positive, *smr*-Positive, n=10	*qacA/B*-Positive, *smr*-Negative, n=10	*qacA/B*-Negative, *smr*-Positive, n=10	*qacA/B*-Negative, *smr*-Negative, n=10	Total
**MRSA**	2	5	6	6	19
**Community-Associated**	6	8	5	8	27
**Community-Onset-Healthcare Associated (1)**	3	1	4	2	10
**Nosocomial (2)**	1	1	1	0	3
**Invasive Infection (3)**	3	2	6	3	14
**Skin and Soft Tissue Infection**	6	8	3	6	23
**Bacteremia/ Central Line Associated Bloodstream Infection**	2	1	2	1	6
**Surgical Site Infection**	1	0	1	1	3
**Deep Abscess**	0	1	0	1	2
**Other**	1	0	4	1	6

Values expressed are number of isolates in each category.

References

1. Hulten KG, Kaplan SL, Gonzalez BE, Hammerman WA, Lamberth LB, Versalovic J, Mason EO, Jr. 2006. Three-year surveillance of community onset health care-associated staphylococcus aureus infections in children. Pediatr Infect Dis J 25:349-53.
2. Hulten KG, Kaplan SL, Lamberth LB, Slimp K, Hammerman WA, Carrillo-Marquez M, Starke JR, Versalovic J, Mason EO, Jr. 2010. Hospital-acquired Staphylococcus aureus infections at Texas Children's Hospital, 2001-2007. Infect Control Hosp Epidemiol 31:183-90.

3. McNeil JC, Hulten KG, Mason EO, Kaplan SL. 2017. Impact of Health Care Exposure on Genotypic Antiseptic Tolerance in Staphylococcus aureus Infections in a Pediatric Population. Antimicrob Agents Chemother 61.

Supplemental Figure. Results of Catheter Disinfection Assay following Exposure to 5,000 µg/ml (0.5%) CHG-Isopropanol Combinations. ME after exposure to 0.5% CHG-0.7% isopropanol (**Panel A**), 0.5% CHG-7% isopropanol (**Panel B**) and 0.5% CHG-70% isopropanol (**Panel C**).

*Rank sum (2 category comparison) p= 0.01-0.05
**Rank sum (2 category comparison) p<0.01
†Kruskal Wallis (4 category comparison P=0.01-0.05
‡Kruskal Wallis (4 category comparison), p<0.01

SA

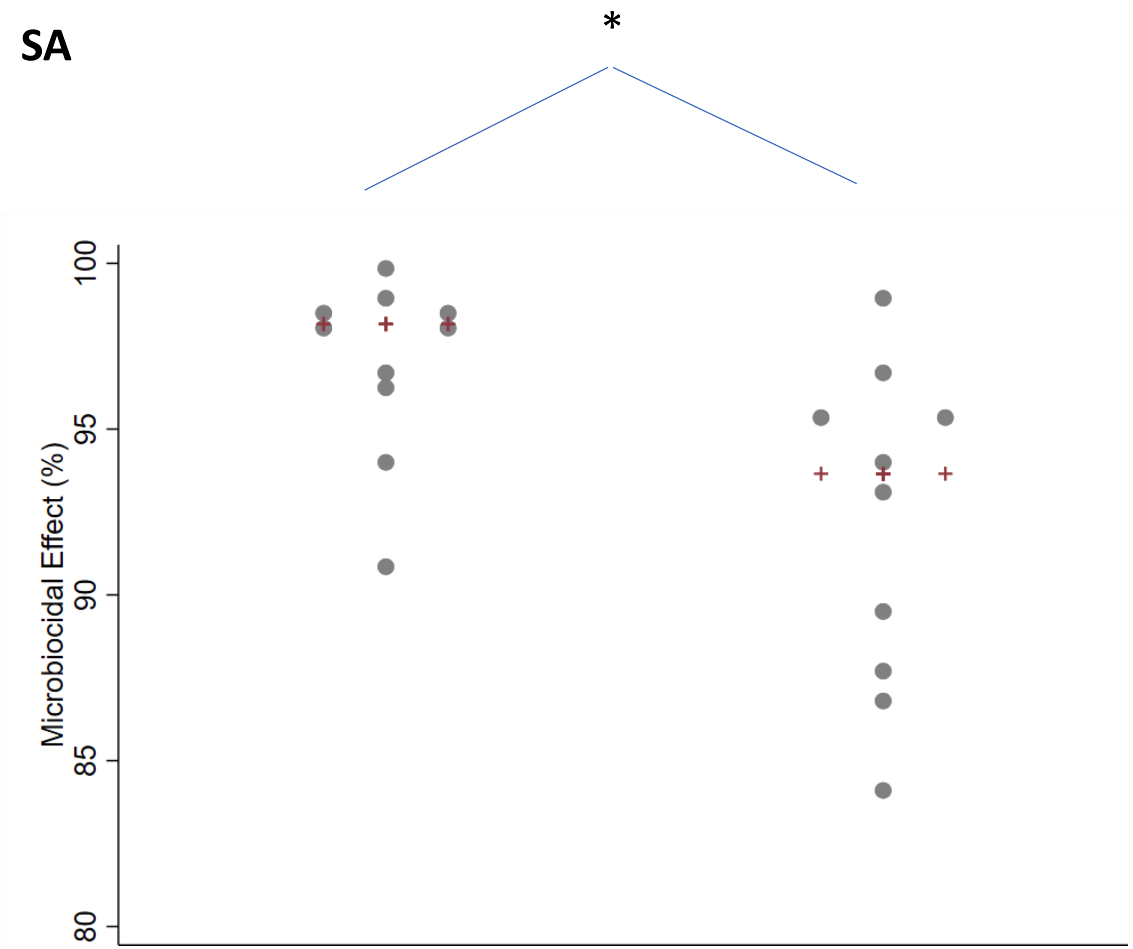

qacA/B	**-**	**+**
smr	**-**	**+**

5,000 µg/ml (0.5%) CHG-0.7% Isopropanol

SB

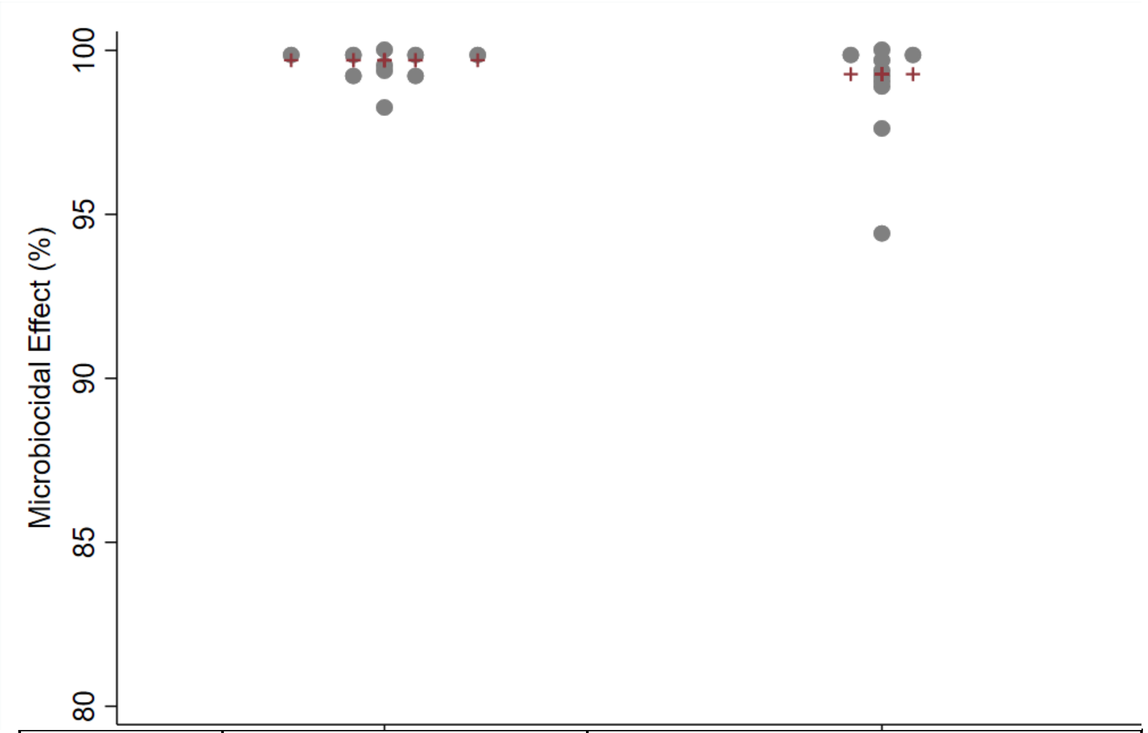

qacA/B	**-**	**+**
smr	**-**	**+**

5,000 µg/ml (0.5%) CHG-7% Isopropanol

Supplemental Figure. Results of Catheter Disinfection Assay following Exposure to 5,000 µg/ml (0.5%) CHG-Isopropanol Combinations. ME after exposure to 0.5% CHG-0.7% isopropanol (Panel A), 0.5% CHG-7% isopropanol (Panel B) and 0.5% CHG-70% isopropanol (**Panel C**).

*Rank sum (2 category comparison) p= 0.01-0.05
**Rank sum (2 category comparison) p<0.01
†Kruskal Wallis (4 category comparison P=0.01-0.05
‡Kruskal Wallis (4 category comparison), p<0.01

SC

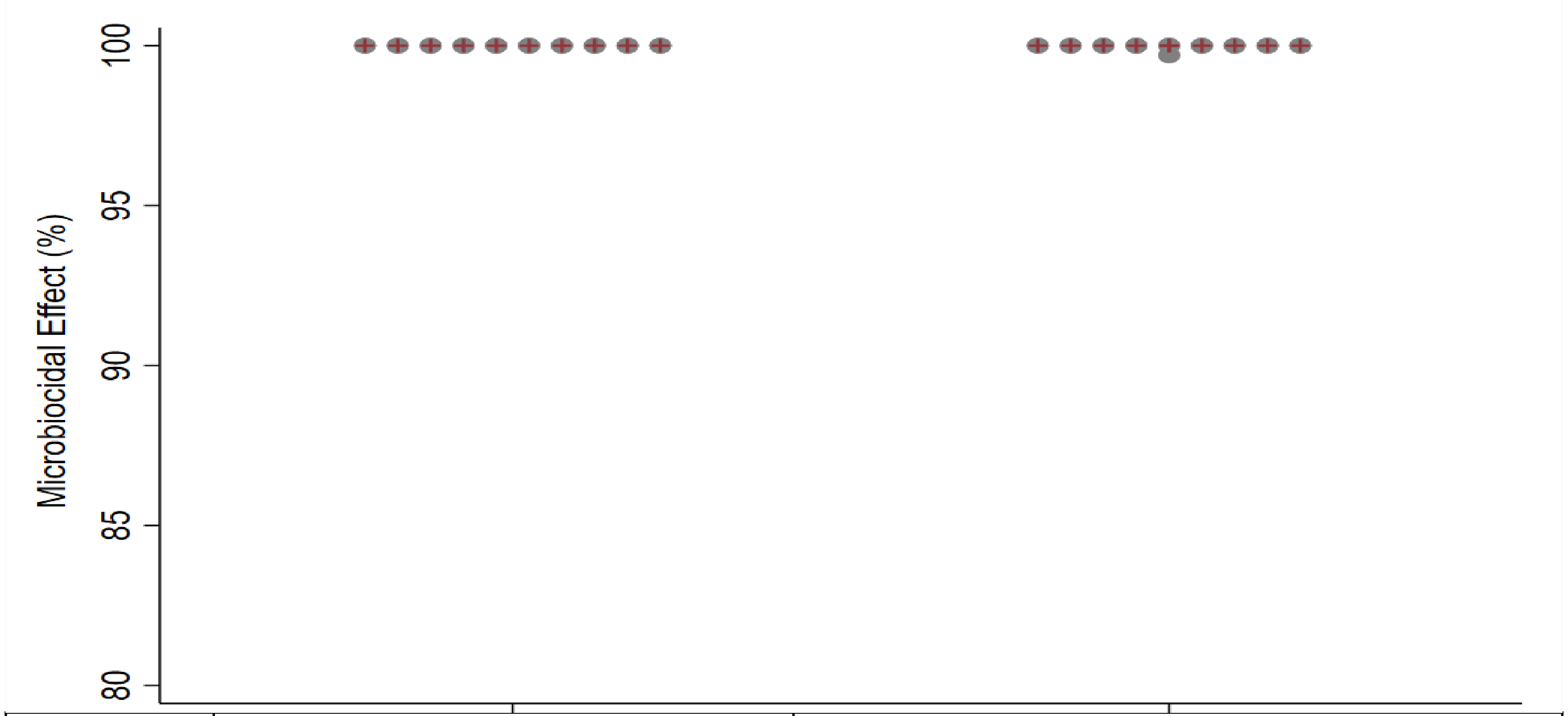

qacA/B	-	+
smr	-	+

5,000 µg/ml (0.5%) CHG-70% Isopropanol
